# Active Aberration Correction with Adaptive Coefficient SPGD Algorithm for Laser Scanning Confocal Microscope

**DOI:** 10.3390/s22103755

**Published:** 2022-05-15

**Authors:** Kunhua Zhou, Zhizheng Wu, Tianyu Zhang, Feng Li, Azhar Iqbal, Suresh Sivanandam

**Affiliations:** 1 Department of Precision Mechanical Engineering, Shanghai University, Shanghai 200444, China; zkh2020@shu.edu.cn (K.Z.); zty759226022@shu.edu.cn (T.Z.); 2 School of Optical-Electrical and Computer Engineering, University of Shanghai for Science and Technology, Shanghai 200093, China; lifenggold@163.com; 3 Dunlap Institute for Astronomy and Astrophysics, University of Toronto, Toronto, ON M5S 3H4, Canada; az.iqbal@utoronto.ca (A.I.); suresh.sivanandam@utoronto.ca (S.S.)

**Keywords:** laser scanning confocal microscope, SPGD, adaptive optics, aberrations, depth imaging

## Abstract

A laser scanning confocal microscope (LSCM) is an effective scientific instrument for studying sub-micron structures, and it has been widely used in the field of biological detection. However, the illumination depth of LSCMs is limited due to the optical aberrations introduced by living biological tissue, which acts as an optical medium with a non-uniform refractive index, resulting in a significant dispersion of the focus of LSCM illumination light and, hence, a loss in the resolution of the image. In this study, to minimize the effect of optical aberrations, an image-based adaptive optics technology using an optimized stochastic parallel gradient descent (SPGD) algorithm with an adaptive coefficient is applied to the optical path of an LSCM system. The effectiveness of the proposed aberration correction approach is experimentally evaluated in the LSCM system. The results illustrate that the proposed adaptive optics system with an adaptive coefficient SPGD algorithm can effectively reduce the interference caused by aberrations during depth imaging.

## 1. Introduction

Nondestructive techniques such as the use of a laser scanning confocal microscope (LSCM) [1,2,3] provide the feasibility of investigating the characterization of biological cells. Scanning can be performed in the lateral as well as in the axial directions, and three-dimensional images of the sample can be generated in this way [2]. However, optical aberrations limit LSCMs to thin samples and have prevented their application to thick specimens [3]. In this paper, an image-based adaptive optics (AO) system with a fast convergence speed is designed for LSCMs to reduce the adverse effects of aberrations and improve imaging quality.

If the aberration can be measured, a wavefront modulator can be employed to introduce compensatory distortion and minimize the net aberration before image formation. Commonly, these systems require reference light sources to measure aberrations, and the light emitted by such a “guide star” accumulates aberrations while traversing the sample and instrument before reaching a wavefront sensor. Fluorescent beads, structures labeled with fluorescent proteins in tissues, and fluorescent protein centrosomes are usually used as reference beacons to measure the aberrations directly and to improve the signal and contrast of images [4,5,6]. However, the method of the artificial introduction of guide stars is relatively complex, and most of these guide stars such as fluorescent proteins are difficult to fix in the sample space. In direct wavefront measurement, the performance relies heavily on the intensity of the light from the samples, which is attenuated exponentially with increasing depth because of scattering in biological tissues. When using Shack–Hartmann wavefront sensing, the scattering will not only limit the amount of photons delivered by the guide star but will also increase the background noise of neighboring guide stars. Both effects reduce the signal-to-noise ratio (SNR) for wavefront measurements. Fluorescent nanodiamonds as new contrast agents exhibit better optical and chemical properties; however, there are still challenges that limit their short- and long-term interactions with living organisms. These include colloidal stability, the specific targeting of structures in biological samples, and brightness against auto-fluorescence [7].

Instead of measuring phase aberrations directly, image-based aberration correction methods without directly using the wavefront sensor carry out aberration correction from a set of image measurements that are obtained when intensity or phase perturbations are introduced into the system [8]. Wavefront-sensorless AO methods are commonly used because they require no extra hardware for direct wavefront sensing and, hence, allow for simpler optical designs and avoid non-common-path sensing errors. A range of wavefront-sensorless AO schemes exist, such as modal [9], pupil segmentation zonal [10], deep learning [11], and blind searching algorithm methods [12,13,14,15,16,17,18,19]. When based on the modal method, the correction speed of the system is fast, but the performance of the control method depends on an accurate mathematical model, and the range of correcting aberrations is limited. The method based on pupil segmentation can detect phase distortion, but it needs to go through complex calculations and requires high image contrast. The recent approaches based on deep learning promise accurate phase retrieval results at fast processing speeds; however, the aberration estimation algorithm needs a bead or a guide star to produce the intensity of images for computation [11]. The blind searching algorithm methods are easy to implement and have a wide range of applications, which mainly use a suitable optimization algorithm, such as the genetic algorithm (GA) [12], particle swarm optimization (PSO) [13], simulated annealing (SA) [14,15], and stochastic parallel gradient descent (SPGD) [16,17,18,19], to iteratively adjust the wavefront until a certain image metric is optimized.

The stochastic parallel gradient descent algorithm is considered in this optics system due to its characteristics such as easy implementation and strong robustness against phase fluctuations. In 1997, Vorontsov, M.A. et al. [16,17] of the U.S. Army Laboratory first proposed the stochastic parallel gradient descent algorithm. Since then, this algorithm has been widely applied in the field of adaptive optics [18,19]. However, the standard SPGD algorithm requires a large number of iterations to obtain the optimal solution, which may lead to a poor real-time performance in biological imaging. In this paper, an SPGD algorithm with an adaptive gain coefficient, which updates the gain coefficient according to the phase of the iteration to increase convergence speed, is proposed for the adaptive optics system of LCSMs, and the effectiveness is experimentally evaluated by conducting depth imaging of a stained leech specimen.

## 2. Adaptive Coefficient SPGD Algorithm for the LSCM System

**Figure 1:**
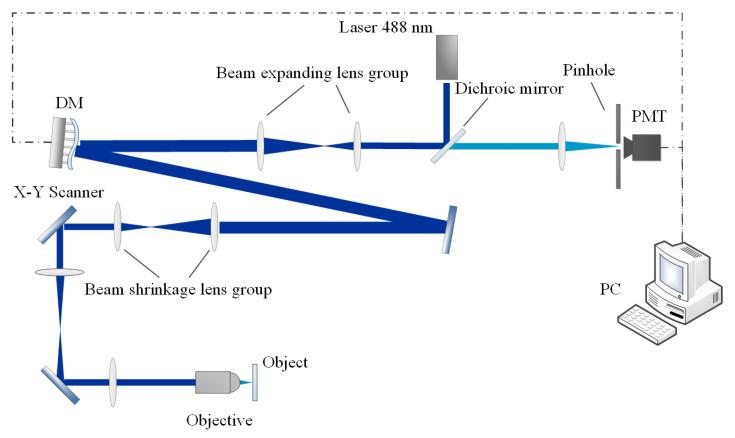
Structure of the AO system for LSCM.

A simplified diagram of a laser scanning confocal microscope with the image-based adaptive optics system is shown in Figure 1. The aberration correction module is located between the excitation laser and the scanning module. A Micro-Electro Mechanical System (MEMS) deformable mirror (DM) is utilized as the wavefront corrector in this system due to its advantages of having an overall light weight, fast response speed, and continuous smooth mirror. The laser beam enters the scanning module through the reflection of the dichroic mirror, the deformable mirror, and the plane mirror. The reflected fluorescence shares a part of the optical path with the incident laser and focuses on the pinhole before the photomultiplier tube (PMT) detector through the dichroic mirror. The computer converts the collected signal of the PMT into an image and controls the deformable mirror according to a certain performance metric of the image. The deformable mirror changes the shape of the mirror surface with different strokes of the internal drivers to change the local optical path difference of the beam reflected by the mirror [20].

In the adaptive optics system, a performance metric, which is supposed to reach the expected extreme value, is used in the algorithm to make the judgment for the completion of the optimization. The performance metric is normally selected to meet the following requirements: it should be obtained easily and be related to the aberration; additionally, the metric of the image collected in the confocal microscope imaging system needs good robustness to background interference.

The grayscale variance value [21], the sharpness [22], and the brightness [23] of the image have been proven to perform well as performance metrics in the image-based adaptive optical system. In this paper, the grayscale variance value of the image, which can reflect the degree of dispersion of the image gray-level distribution, is selected because of its robustness for sample aberration compensation. The metric J can be written as

(1) $$J=\sqrt{\frac{1}{MN}{\sum_{x,y}\left(I\left(x,y\right)-\bar{I}\right)}^{2}}$$

where MN represents the product of the width and height pixels, I(x,y) represents the grayscale value of the corresponding coordinate point in the image, and $\bar{I}$ is the average grayscale of the image.

SPGD is a blind optimization heuristic search algorithm, which is based on the principle of the gradient descent algorithm. The iterative calculation formula of the gradient descent can be written as the following equation:

(2) $$x^{\left(k+1\right)}=x^{\left(k\right)}-\lambda \nabla f[x^{\left(k\right)}]$$

where $x^{\left(k\right)}$ is the input signal at the k-th iteration, λ is the step size, and $\nabla f[x^{\left(k\right)}]$ is the gradient of the metric performance. When we apply the above formula to the DM control system, we can obtain

(3) $$u_{j}^{\left(k+1\right)}=u_{j}^{\left(k\right)}+\varepsilon \frac{\partial J}{\partial u_{j}}$$

where ε is the coefficient that affects the direction and speed of the optimization, and $u_{j}^{\left(k\right)}$ is the control signal applied by the j-th driver of the deformable mirror at the k-th iteration. The value of $\frac{\partial J}{\partial u_{j}}$, which cannot be calculated directly, should be estimated by the perturbation of the control input and related performance metric variances. In the AO system, the estimate of $\frac{\partial J}{\partial u_{j}}$ can be written as [24]

(4) $$\frac{\partial J}{\partial u_{j}}\approx \frac{\delta J\delta u_{j}}{\sigma^{2}}$$

where σ is the amplitude of the random perturbation, and $\delta u_{j}$ is the independent perturbation applied to the k-th control signal. δJ is the change of metric caused by perturbation and can be written as the following equation when perturbed bilaterally:

(5) $$\delta J=\frac{1}{2}\left[J(u+\delta u)-J(u-\delta u)\right]$$

The optimized formula of the SPGD algorithm can be written as

(6) $$u_{j}^{\left(k+1\right)}=u_{j}^{\left(k\right)}+\gamma \delta J^{\left(k\right)}\delta u_{j}^{\left(k\right)}$$

where $\gamma =\varepsilon /\sigma^{2}$ is the gain coefficient. For the method of fixed gain coefficient, it is necessary to determine a more appropriate coefficient through repeated experiments, as it needs a large number of optimization iterations. In this paper, an adaptive method that depends on the change phase of the metric is applied to update the gain coefficient. The gain coefficient after k iterations is designed as

(7) $$\gamma^{\left(k+1\right)}=\gamma^{\left(k\right)}+\alpha \left(\gamma_{0}-\gamma^{\left(k\right)}\right)+\beta^{\left(k\right)}H^{\left(k\right)}$$

and

(8) $$\{\begin{array}{l} \beta^{\left(k\right)}=\xi \left|sign(\Delta J_{+}^{\left(k\right)})-sign(\Delta J_{-}^{\left(k\right)})\right|\\ H^{\left(k\right)}=\left|\delta J^{\left(k\right)}\right|\cdot \sum_{l=1}^{L}\left|J^{\left(k\right)}-J^{\left(k-l\right)}\right|\cdot \exp\left(-\frac{1}{J_{0}-J^{\left(k\right)}}\right) \end{array}$$

Equation (7) is adopted to ensure that the function converges, where $\gamma_{0}$ is the value that γ eventually converges at and α is the fixed coefficient that determines the rate of convergence. $\beta^{\left(k\right)}H^{\left(k\right)}$ constitute the adaptive gain term. $\Delta J_{+}^{\left(k\right)}$ and $\Delta J_{-}^{\left(k\right)}$ represent the change of performance metric J under positive and negative perturbations, respectively. $\beta^{\left(k\right)}$ will be zero when $\Delta J_{+}^{\left(k\right)}$ and $\Delta J_{-}^{\left(k\right)}$ have the same sign, and it will be 2ξ when $\Delta J_{+}^{\left(k\right)}$ and $\Delta J_{-}^{\left(k\right)}$ have different signs; ξ is a constant. $H^{\left(k\right)}$ is used to identify the change phase of the performance metric in the adaptation process, and it depends on the amount of change in the current metric caused by the parallel perturbation, the total change of metric in the recent l iterations, and the difference between the current metric J and the desired metric $J_{0}$. The value of $H^{\left(k\right)}$ is larger when the current change slope of the metric is greater or the distance to the desired optimal value is farther away, and the value of $H^{\left(k\right)}$ tends to be zero when the metric changes little in the recent l iterations and is close to $J_{0}$. Because of the existence of $H^{\left(k\right)}$, the algorithm can effectively reduce the probability of falling into local extreme values and achieve good convergence performance in practical applications.

**Figure 2:**
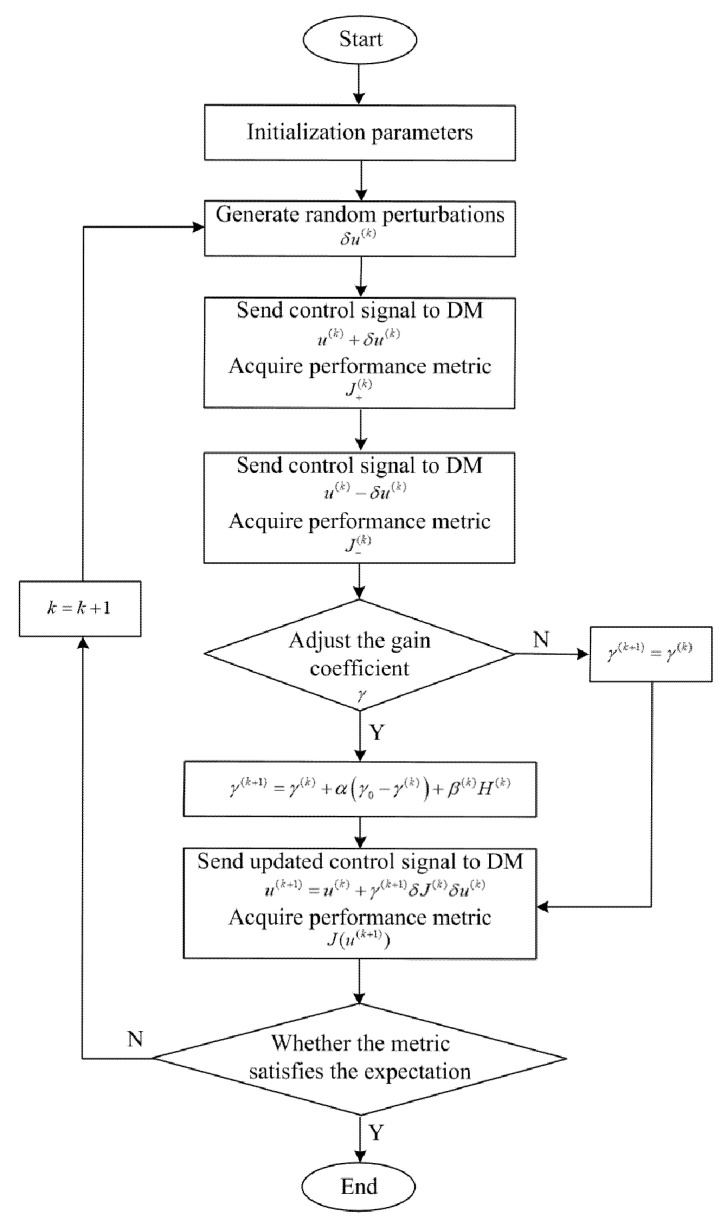
Flow of SPGD algorithm for LSCM.

With the adaptive coefficient, the algorithm can first approach the optimal solution with a larger gain coefficient when seeking the extreme value of the objective function, and then it can adjust to a smaller one to improve the accuracy. The flow of the adaptive coefficient SPGD algorithm for LSCM is shown in Figure 2.

## 3. Experiment and Results

**Figure 3:**
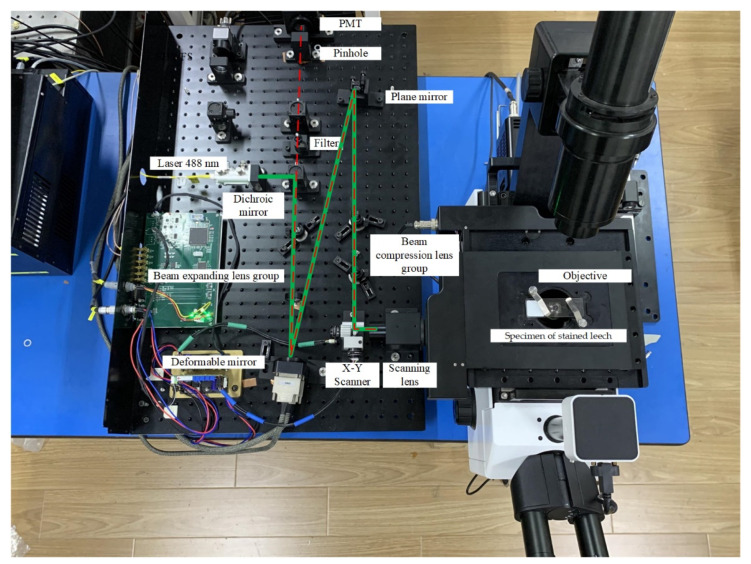
Photograph of the AO imaging system for LSCM.

The experimental devices are shown in Figure 3. A laser with a wavelength of 488 nm, which is collimated and outputs through a single-mode fiber, is used as the excitation light source. The diameter of the output spot is 0.7 mm. The laser is reflected by a dichroic mirror (ZT405/488/561/640rpcv2-UF1, CHROMA, Bellows Falls, VT, USA) into a beam-expanding lens group consisting of achromatic lenses with focal lengths of 10 mm (AC080-010-A, THORLABS, Newton, NJ, USA) and 125 mm (THORLABS, AC254-125-A). The expanded beam spot size is about 9 mm, and it is reflected by a deformable mirror (DM-69, ALPAO, Montbonnot-Saint-Martin, France), with sufficient linearity and repeatability properties, and by another mirror (THORLABS, BB05-E02) into the beam-compression lens group, which consists of achromatic lenses with focal lengths of 75 mm (THORLABS, AC127-075-A) and 30 mm (THORLABS, AC127-30-A). The beam spot size after compression is about 3.6 mm. The laser beam enters the scanning lens (CDGM, Rochester, NY, USA) through the scanning galvanometer (SUNNY, S-8107, SUNNY, Beijing, China), and finally, it enters the objective (Plan N 40x/0.65, OLYMPUS, Tokyo, Japan) of the inverted fluorescence microscope.

The fluorescence of the sample excited by the excitation light returns along the original optical path and transmits through the dichroic mirror. The filter (CHROMA, 488 nm) behind the dichroic mirror can filter out the residual excitation laser light reflected by the sample. The fluorescent light focuses on the pinhole (THORLABS, P50D) through the pinhole lens and is ultimately detected by the photomultiplier tube detector (H10721-20, HAMAMATSU, Hamamatsu, Japan) through an optical fiber.

The test object is a stained leech specimen. Firstly, the fixed coefficient SPGD algorithm is adopted to control the deformable mirror for the unknown aberration elimination. Then, the proposed adaptive coefficient SPGD algorithm is further evaluated, and the experimental results are compared with the algorithm with a fixed coefficient. The experimental parameters in both algorithms are selected as shown in Table 1.

**Figure 4:**
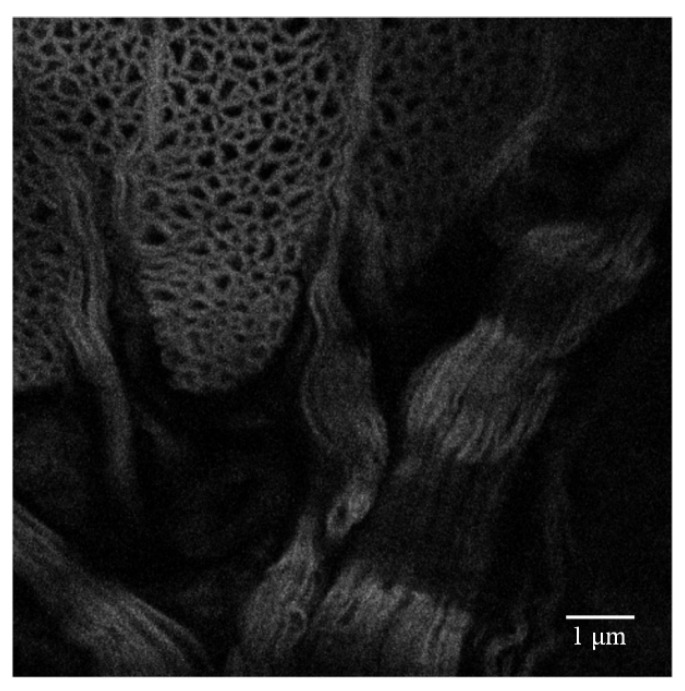
Image of the leech specimen at −0.0415 mm in the *z*-axis direction.

**Figure 5:**
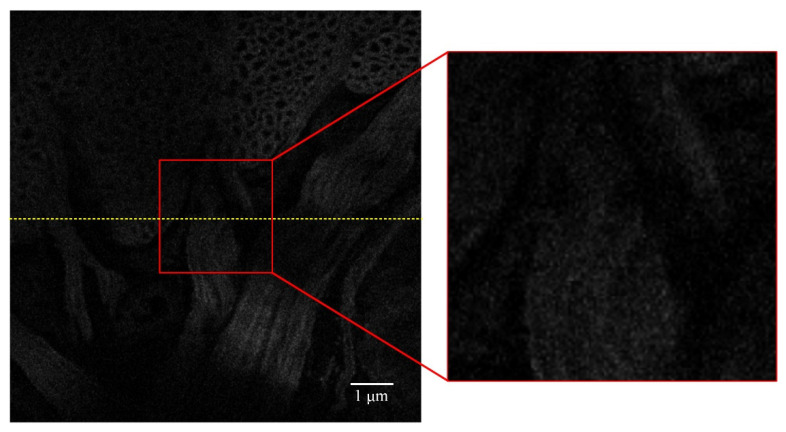
Initial imaging result of the specimen at −0.0815 mm in the *z*-axis direction, and the grayscale variance value is about 6.0.

The microscope displacement platform is located at −0.0415 mm in the Z-axis direction to obtain a clearer image of the leech with the Olympus 40× objective lens, as shown in Figure 4. Then, the displacement platform of the inverted fluorescence microscope is adjusted to move in the Z-axis direction. When the displacement platform is located at −0.0815 mm, where the imaging depth is around 40 μm, it can be seen in Figure 5 that, due to aberrations, the fluorescent signal is weak, and the image becomes blurred. It is difficult to see the details in the partial enlargement area clearly.

**Figure 6:**
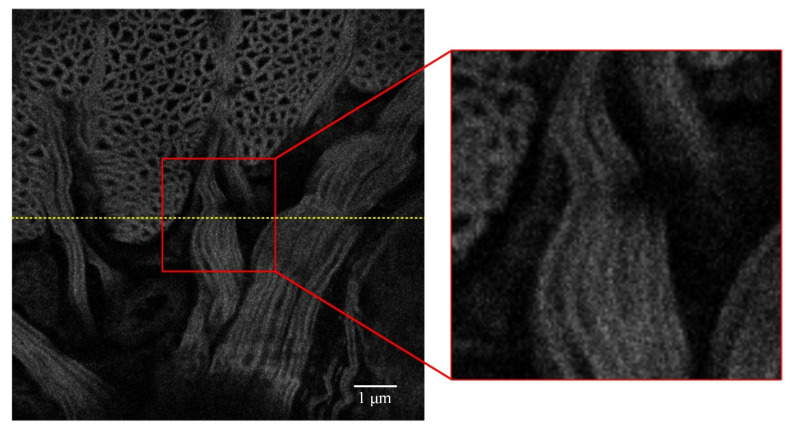
Imaging result after correction with 100 iterations of the fixed coefficient SPGD algorithm, and the grayscale variance value is about 8.9.

**Figure 7:**
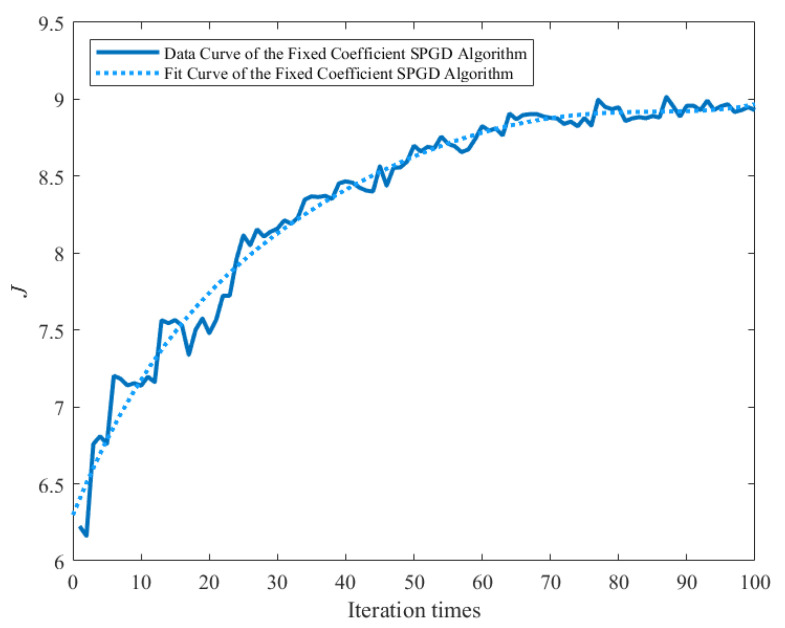
Metric convergence curve of the fixed coefficient SPGD algorithm.

When the fixed coefficient SPGD algorithm is used to control the deformable mirror to correct unknown aberrations, a relatively clear image with a strong fluorescence signal is obtained, as shown in Figure 6. The image quality is significantly improved, and the details in the image of the partial enlargement area at the same location can be distinguished well. The performance metric convergence curve of the fixed coefficient SPGD algorithm is shown in Figure 7. The metric of the fixed coefficient SPGD algorithm is close to the convergence value of about 8.9 after 70 iterations, which takes around 50 s.

**Figure 8:**
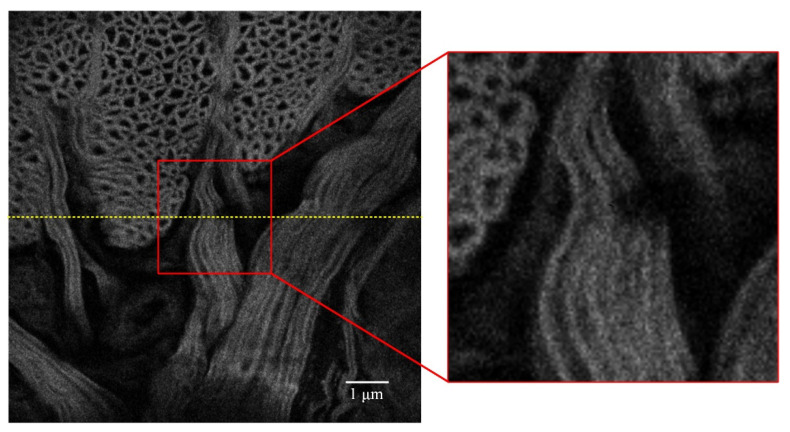
Imaging result after correction with 100 iterations of the adaptive coefficient SPGD algorithm, and the grayscale variance value is about 9.0.

**Figure 9:**
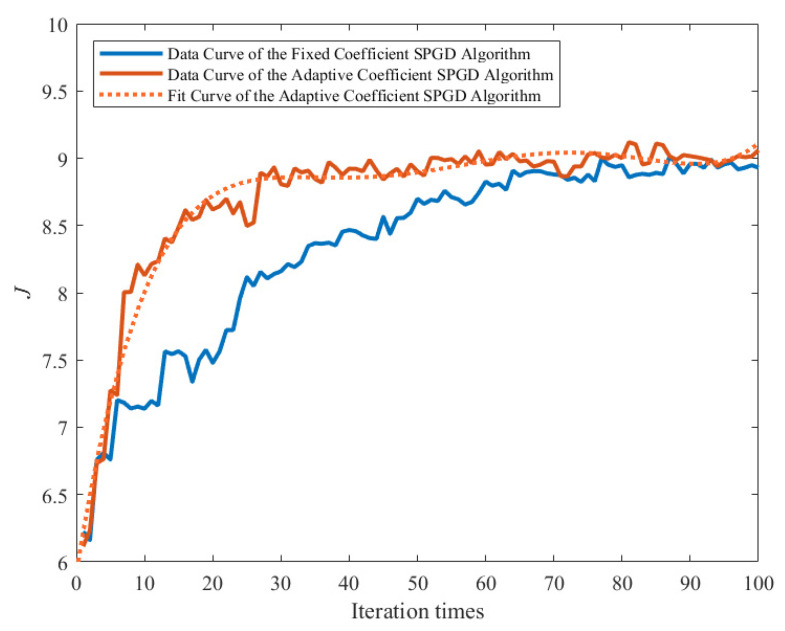
Metric convergence curve of the adaptive coefficient SPGD algorithm.

**Figure 10:**
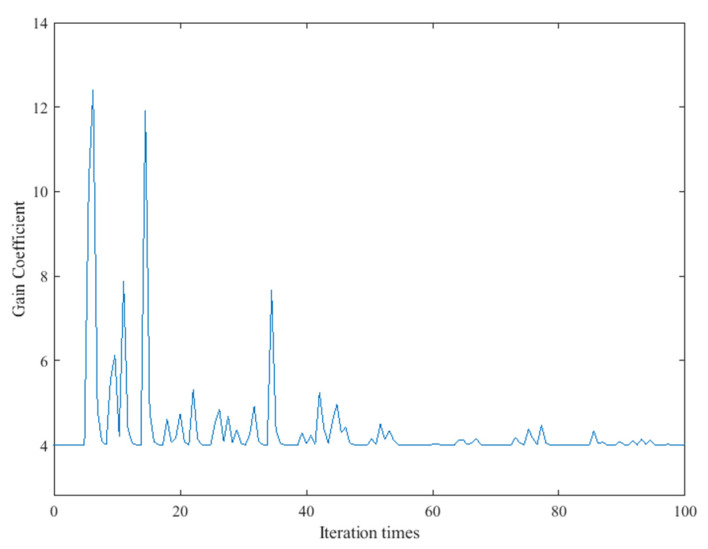
Curve of the update gain coefficient of the SPGD algorithm applied to the LSCM system.

**Figure 11:**
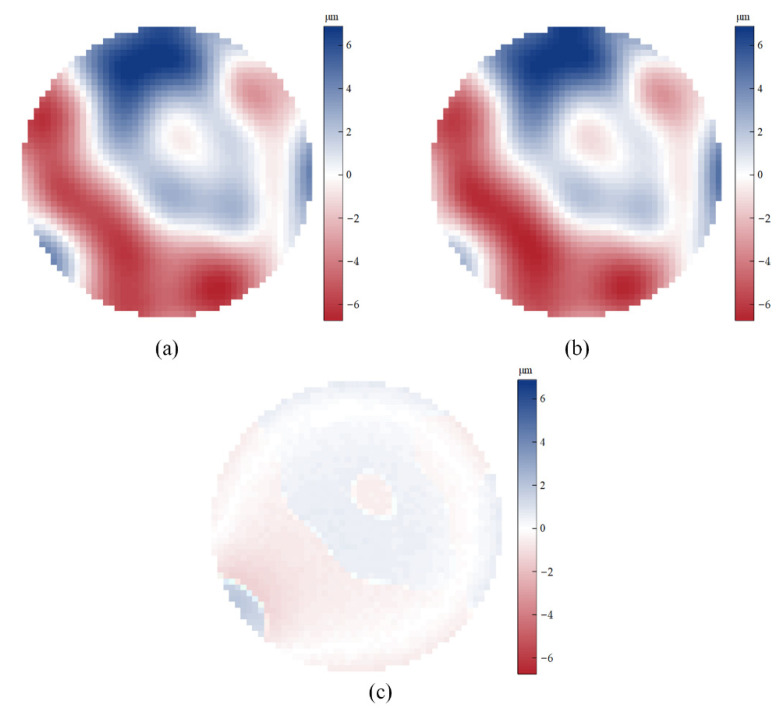
Different shapes of the deformable mirror: (**a**) shape of the deformable mirror controlled by the fixed coefficient SPGD algorithm; (**b**) shape of the deformable mirror controlled by the adaptive coefficient SPGD algorithm; (**c**) difference phase map of two shapes of the deformable mirror.

Then, the control signal of the deformable mirror is reset, and the adaptive coefficient SPGD algorithm is further tested to obtain the resulting image shown in Figure 8. The performance metric convergence curve of the adaptive coefficient SPGD algorithm is shown in Figure 9. It converges at the value of 9.0 after 40 iterations, which takes around 28 s. The curve of the updated gain coefficient of the SPGD algorithm is shown in Figure 10. Because the coefficient update needs to consider the results from the recent five iterations, the coefficient starts the update in the sixth iteration. Combined with Figure 9 and Figure 10, the gain coefficient adaptively increases to make the metric approach the optimal value quickly when the value of the performance metric is small. It varies greatly at the beginning and then converges to the value of 4. Since the initial coefficient value of the adaptive coefficient SPGD algorithm is smaller than that of the fixed coefficient SPGD algorithm, the proposed adaptive coefficient SPGD algorithm can obtain a better performance metric value. The surface shapes of the deformable mirror after iterations of the SPGD algorithms with a fixed coefficient and an adaptive coefficient are shown in Figure 11a,b, respectively, and they represent the degree of each corrected aberration. It can be seen that the aberration corrected by the adaptive coefficient SPGD algorithm is relatively better, and the difference phase map is shown in Figure 11c.

**Figure 12:**
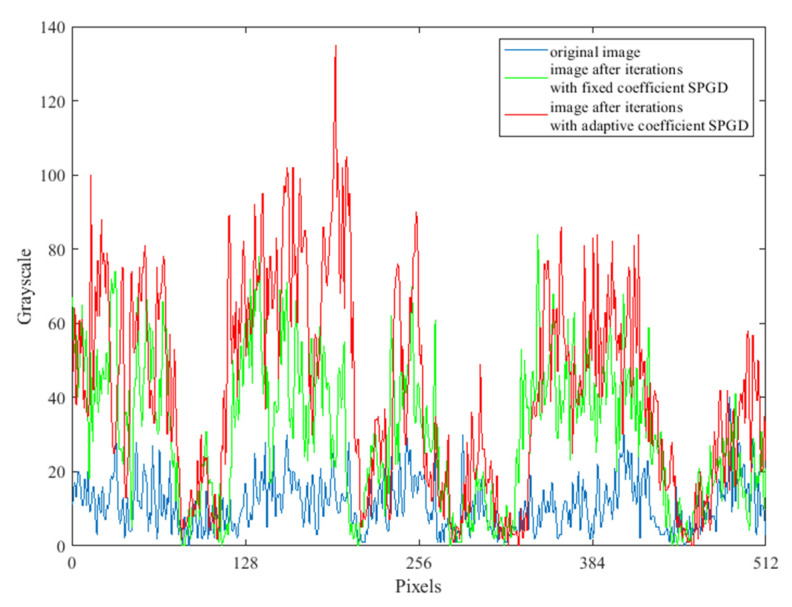
Grayscale graphs at the yellow line of the original image and two images after aberration correction.

The grayscale value curves at the yellow lines in Figure 5, Figure 6, and Figure 8 are shown in Figure 12. The grayscale values of the original image are low and concentrated. When the two SPGD algorithms are adopted to correct aberrations, the grayscale values of the images are high and widely distributed, and among them, the grayscale values of the image obtained after iterations with the adaptive coefficient SPGD algorithm are relatively high and more widely distributed, which means that the quality of the image is better.

The proper settings of the initial values of the parameters in Equations (7) and (8) could affect the final convergence performance of the SPGD algorithm, which can normally be optimized based on enough experiment testing data or experience. In the future, the optimal selection approach of the initial parameters will be further studied, e.g., combining the AI algorithm to achieve generalized rules for different applications.

## 4. Conclusions

In this paper, an image-based AO is applied for aberration correction in a laser scanning confocal microscope system. The SPGD algorithm with an adaptive coefficient is used to control the DM to compensate for the unknown wavefront aberration caused by the non-uniform refractive index of samples. Unlike the traditional SPGD algorithm, the gain coefficient of the optimized SPGD algorithm updates according to the phase of the iteration. Evaluated through a series of imaging experiments, the proposed SPGD algorithm can not only reduce the number of iterations but can also improve the accuracy, and it performs well in LSCM depth imaging.

## Figures and Tables

**Table 1:** Parameters used in the experiments.

Parameters	$\left|\delta u_{j}\right|$	$\gamma_{0}$	α	ξ	l	$J_{0}$
Fixed coefficient SPGD algorithm	0.005	5	--	--	--	--
Adaptive coefficient SPGD algorithm	0.005	4	0.9	15	5	10

## Data Availability

The data presented in this study are available on request from the corresponding author.
